# Numerical and Experimental Research of the Plastic Forming Process of Hastelloy X Alloy Sheets Using Elastomeric and Steel Tools

**DOI:** 10.3390/ma17225473

**Published:** 2024-11-09

**Authors:** Krzysztof Żaba, Maciej Balcerzak, Tomasz Trzepieciński, Łukasz Kuczek, Vit Nowak, Jarosław Mizera, Ryszard Sitek

**Affiliations:** 1 Department of Metal Working and Physical Metallurgy of Non-Ferrous Metals, Faculty of Non-Ferrous Metals, AGH University of Krakow, al. Adama Mickiewicza 30, 30-059 Cracow, Poland; krzyzaba@agh.edu.pl (K.Ż.); balcerzak@agh.edu.pl (M.B.); lukasz.kuczek@agh.edu.pl (Ł.K.); 2 Department of Manufacturing Processes and Production Engineering, Faculty of Mechanical Engineering and Aeronautics, Rzeszów University of Technology, al. Powstańców Warszawy 8, 35-959 Rzeszów, Poland; tomtrz@prz.edu.pl; 3 Department of Manufacturing Technology, Faculty of Mechanical Engineering, Czech Technical University in Prague, Technická 4, 166 07 Prague, Czech Republic; vit.novak@fs.cvut.cz; 4 Faculty of Materials Science and Engineering, Warsaw University of Technology, Wołoska 141, 02-507 Warsaw, Poland; jaroslaw.mizera@pw.edu.pl

**Keywords:** sheets plastic forming, Hastelloy X, elastomeric punches, numerical modeling, optical 3D scanning

## Abstract

The results of experimental and numerical studies of plastic forming of sheets made of the difficult-to-deform Hastelloy X, a nickel-based alloy with a thickness of 1 mm, using layered elastomeric punches and steel dies, are presented in this publication. The elastomeric punches were characterized by hardness in the range of 50–90 Shore A, while the dies were made of 90MnCrV8 steel with a hardness of over 60 HRC. The principle of operating the stamping die was based on the Guerin method. The finite-element-based numerical modeling of the forming process for various configurations of polyurethane inserts was also carried out. The results obtained from numerical modeling were confirmed by the results of experimental tests. The drawpieces obtained through sheet forming were subjected to geometry tests using optical 3D scanning. The results confirmed that in the case of forming difficult-to-deform Hastelloy X, Ni-based alloy sheets, the hardness of the polyurethane inserts significantly affected the geometric quality of the obtained drawpieces. Significant nonuniform sheet metal deformations were also found, which may pose a problem in the process of designing forming tools and the technology of the plastic forming of Hastelloy X, Ni-based alloy sheets.

## 1. Introduction

Nickel-based superalloys have many unique functional properties, such as heat resistance, creep resistance, corrosion resistance, and high strength at ambient temperature [1,2]. They are used in the construction of machines and devices operating in extreme conditions, such as high temperatures, variable loads, and aggressive corrosive environments of gases and harmful chemical compounds [3,4]. A special feature of Ni-based superalloys is the ability to work continuously at temperatures up to about 1250 °C and periodically even up to 1400 °C. Due to their properties, nickel alloys are widely used in the aviation industry for the construction of jet engine parts and gas turbines and in the nuclear, chemical, and petrochemical industries. Components made of nickel alloys are usually manufactured using sheet metal forming methods [5].

Forming of nickel alloy sheet metals, mainly used in the aviation and space industry, is a complicated process that requires multi-stage processing [6]. Ni-based alloys are difficult to deform at room temperature [7]. To obtain the appropriate functional properties of the formed components, it is necessary to simultaneously plan the proper heat treatment. When selecting the parameters for forming superalloys, many technological aspects should be taken into account [8]. Too high values of strains can lead to material cracking, while values that are too low can only cause deformations in the area near the material surface and deterioration of the surface quality of formed components [9]. To obtain a uniform distribution of plastic strains in the entire volume of the material, the forming process must be properly planned and carried out. Otherwise, plastic forming can lead to an inhomogeneous microstructure [10,11]. The forming of difficult-to-deform alloy drawpieces with moderately shallow, recessed parts having simple flanges is accomplished by using elastomeric materials or rubber [12,13]. These materials exert nearly equal pressure on all surfaces of the workpiece when pressed against the metallic die [14]. The Guerin process [15], which uses a rubber punch placed in a rigid box, is commonly used for forming short runs of lightweight metal parts with dies placed on the lower press plate. Although elastomeric stamping tools are simpler in design than traditional tools, their design presents many difficulties to designers [16,17]. When designing a tool for stamping using elastomeric materials, the first step is to analyze whether a given shape of the part can be manufactured using these tools [18]. It is important that the shape is properly defined and does not have sharp edges that could damage the forming tool. Another issue is the bending radii that can be achieved during stamping. The minimum bending angle varies depending on the material being formed and its thickness [19]. An obvious aspect of sheet metal stamping using elastomeric materials is the application of the right force for forming the sheets. The process parameters must be appropriately selected for the formed element, based on the physico-mechanical properties of elastomeric material used for stamping, the material of the formed sheet metal, and the desired shape [18,20].

Elastomeric materials have many advantages when used for the plastic forming of sheet metals. These include significant wear resistance and resistance to chemicals [21]. The development of elastomeric materials has led to research work in which polymer materials have been successfully used as parts of tools for cutting, stamping, bending, or deep drawing [22]. The first elastomeric materials used in plastic forming processes were natural rubber, butadiene-styrene rubber, and silicon rubber. In the case of using butadiene-styrene rubber as a stamping tool, it was observed that it is stiffer at low stresses and is characterized by a more uniform increase in stiffness at high stresses, compared to natural rubber. The next material introduced in industrial applications was polyurethane rubber, which is highly resistant to wear and it is thermally stable [23]. At the same time, this material behaves both as an elastic solid and as a viscoelastic fluid under certain conditions. Currently, due to the above-mentioned advantages, polyurethanes are the most commonly used material in sheet metal forming processes using elastomeric tools [14].

Due to the difficulties in forming high-strength nickel alloys, many researchers have undertaken studies on the forming of this type of material using various forming methods. Sala [15] optimized the Guerin forming process of the EN AW-2024 aluminium alloy fuselage frame belonging to AerMacchi aircraft. The effects of component geometry, stamping velocity, and styrene-butadiene-rubber pad constitutive law on the component quality were investigated. Studies have shown that numerical simulation is able to reveal the potential defects in the formed component and determine technological parameters, such as punch speed and rubber properties, without conducting a trial-and-error approach. Lee et al. [13] investigated the deformation characteristics of the extruded tube to study the effect of process parameters on the deformation of the rectangular EN AW-6061 aluminium alloy tube. The results revealed that to decrease the minimum formable radius of curvature of tubes, the rubber hardness should be decreased. Ramezani et al. [14] analyzed experimentally the effect of rubber material and punch speed on specimen thinning in flexible punch forming of aluminium sheets. They confirmed that the polyurethane rubber pad forming (RPF) process reduces the sheet thinning in the drawpiece, compared to the conventional forming process. Balcerzak et al. [23] used layered polyurethane-pads to form the embosses in drawpieces made of Inconel 625 Ni-based alloy. The results of FE-based numerical simulations and experiments have shown that the hardness of the elastomeric materials had a significant impact on the quality of the obtained components. Halkacı et al. [24] numerically analyzed the formability of DX56D and Inconel 625 sheets of metal blanks in the RPF process. The drawpieces were successfully formed in 2D axisymmetric simulations with a gain of approx. 50% in CPU time as compared to 3D approaches. Liu and Hua [25] manufactured a metallic bipolar plate of SS304 stainless steel with perfect flow micro-channels through RPF. It was found that the rubber hardness is not an important parameter for the formation of a bipolar plate. In the study [26], polyurethane rubber was adopted as the punch material. The rubber punch material was combined with a reconfigurable multi-point forming die. A significant decrease in wrinkling and springback in the final formed part was achieved. At the same time, the forming costs were reduced by at least 50%.

The use of increased process temperature, especially in the case of difficult-to-deform alloys, contributes to the improvement of material flow. As a result, it is possible to obtain higher drawpieces with better shape accuracy compared to products obtained at room temperature. However, in the case of some nickel alloys, the use of too high forming temperatures may contribute to a reduction in uniform deformation which is important in terms of the possibility of forming sheets using stamping processes, including elastoforming and, consequently, may negatively affect the possibility of forming sheets.

In the case of elastoforming, the working temperature range for a given elastomer must also be taken into account. In the case of polyurethane, its temperature stability is up to 150 °C. These values are significantly lower than the range of plastic deformation in hot or warm conditions, especially in the case of nickel alloys.

An important parameter influencing the process of forming sheet metal using one of the elastomer tools is the shape of this tool. The appropriate shape of the elastomer contributes to the absence of cracks in the product, and the target shape of the product is possible achieved with good accuracy.

In order to limit the variability of the forming process, it is necessary to control the friction coefficient of the sheet and lubrication during the process which allows us to avoid excessive thinning of the material and the possible occurrence of cracking.

This article presents an innovative approach to the use of layered polyurethane inserts in an elastomeric punch. The advantage of using multilayer polyurethane inserts is that in the event of their wear, the possibility of replacing individual layers and greater flexibility of the production process resulting from the possibility of configuring punches with different characteristics. Experimental studies and finite element-based numerical simulations of the forming of Hastelloy X alloy sheets using a Guerin box press forming were carried out. Different configurations of polyurethane rubber inserts with hardness between 50 and 90 ShA were considered. Based on the optical measurements of the drawpieces, sheet thinning, flatness deviations of the drawpieces, and uniformity of forming were determined.

## 2. Materials and Methods

### 2.1. Test Material

The tests were performed using 1 mm thick sheets of Hastelloy X (AMS 5536) nickel-based alloy [27]. Hastelloy X alloy is a nickel–chromium–iron–molybdenum alloy with many alloying elements (Table 1), with anti-corrosion properties and high-temperature resistance.

Hastelloy X alloy can be used in difficult working conditions, such as high temperatures and chemical corrosion environments, which makes it often used in the petrochemical, aerospace, and space industries.

**Figure 1:**
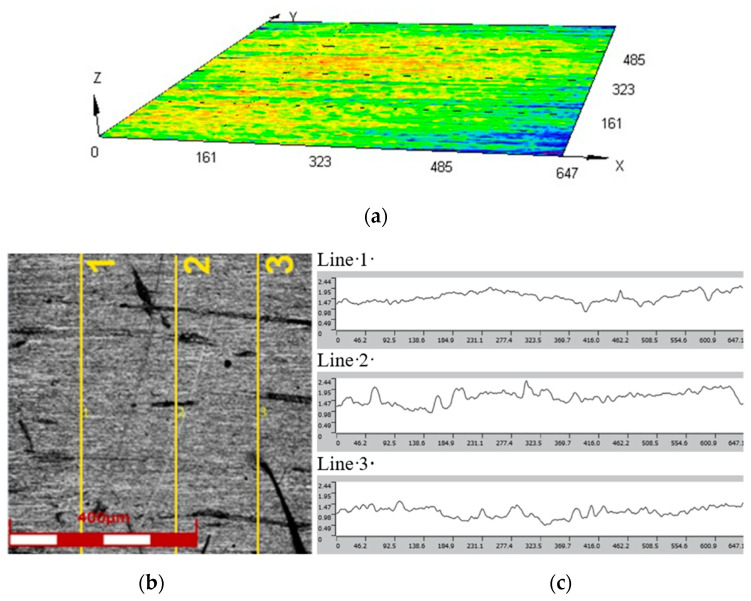
(**a**) Hastelloy X surface topography of Hastelloy X sheet metal in as-received state; (**b**) laser imaging with plotted measurement lines; and (**c**) roughness profiles along lines 1–3.

Hastelloy X alloy contains a number of strengthening phases that affect its mechanical properties and corrosion resistance. The most important strengthening phases are chromium carbide (Cr_7_C_3_), molybdenum carbide (Mo_2_C), and γ′ (nickel-iron) phase. The Cr_7_C_3_ is characterized by the high hardness and corrosion resistance. It is a brittle phase that improves wear resistance. The Mo_2_C is a phase with high hardness and corrosion resistance that improves abrasion and corrosion resistance. The γ′ phase is the most important strengthening phase in Hastelloy X alloy, with high hardness, corrosion resistance, and excellent mechanical properties, including high tensile strength and fatigue resistance [28,29,30]. The LEXT OLS4100 (Olympus, Tokio, Japan) confocal laser microscope, which operates using UV laser light with a wavelength of 405 nm, was used to determine the surface roughness parameters and surface topography (Figure 1). The average values of the basic roughness parameters from three measurements were: Ra = 0.14 μm and Rz = 1.45 μm.

### 2.2. Material Testing of Hastelloy X Alloy

**Figure 2:**
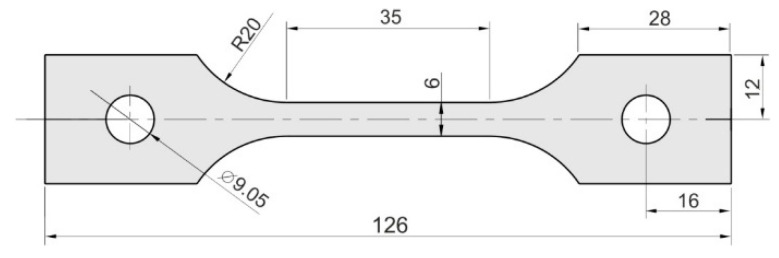
Dimensions (in mm) of samples for tensile test.

The basic mechanical properties of the sheets were determined in a uniaxial tensile test using a Zwick/Roell Z100 testing machine (Zwick/Roell, Ulm, Germany), in accordance with the international standard ISO 6892-1:2020-05 [31]. The strain rate was 0.008 s^−1^. The samples (Figure 2) were prepared by mechanical processing, with the shape and dimensions determined based on the standard [31].

The analysis of sheet anisotropy was performed based on a uniaxial tensile test of samples cut at an angle of 0°, 90°, and 45° relative to the rolling direction of the sheet. The basic test length was set to 25% of the sample elongation, which was determined from the tensile test up to the moment of rupture. A measurement base of 30 mm in length was marked on the sample in the middle of the measurement length. The average thickness value from three measurements was used for the calculation of anisotropy parameters [32]. The following formula was used to calculate the normal anisotropy r-value [33]:

(1) $$r_{x}=\frac{\ln\frac{b}{b_{0}}}{\ln\frac{L_{0}b_{0}}{\mathrm{Lb}}}$$

where b_0_ and b are the sample widths before and after the tensile test, respectively; L_0_ and L are sample lengths before and after the tensile test, respectively; x is the designation of the sampling angle relative to the rolling direction of the sheet.

The mean normal anisotropy coefficient rav was determined according to the relationship [33]:

(2) $$r_{\mathrm{av}=}\;\frac{r_{0}+r_{90}+2r_{45}}{4},$$

where r_0_, r_45_, and r_90_ are the normal anisotropy coefficients determined at 0°, 45°, and 90° relative to the rolling direction, respectively.

The plane anisotropy coefficient was determined according to the relationship [33]:

(3) $$\Delta r=\frac{r_{0}+r_{90}-2r_{45}}{2},$$

The basic material tests using the uniaxial tensile test were supplemented by a formability test [34] performed using the Erichsen 142/40 machine. The diameter of the punch and die was 20 mm and 27 mm, respectively. The rounded punch was pressed at a speed of 5 mm/min into the sheet metal until a crack appeared on its surface. The samples were made for the tests in the form of squares with dimensions of 100 mm × 100 mm. In the formability tests of the sheets, three samples were used for each test condition.

### 2.3. Polyurethane Materials Research Methodology

**Figure 3:**
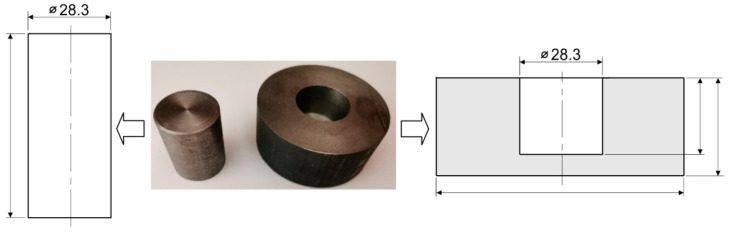
Punch and container used for volumetric compression tests.

Inserts made of commercial polyurethane with hardnesses of 50, 60, 70, 80, and 90 ShA were used for the tests. The hardness measurement was carried out using a Shore A hardness tester. Polyurethane rubber strength tests were also carried out using a volumetric compression test. The volumetric compression test of polyurethane rubber was performed on a Zwick/Roell Z100 testing machine to determine the force necessary to compress the elastomeric material. For this purpose, a specially prepared tool was used in the form of a punch and a container shown in Figure 3. Cylindrical samples with a diameter of ϕ = 28 mm and height h = 13 mm were used. The speed of the testing machine crosshead was 12 mm/min and the punch displacement was 1.5 mm.

### 2.4. Forming Methodology for Hastelloy X Alloy Sheets

**Figure 4:**
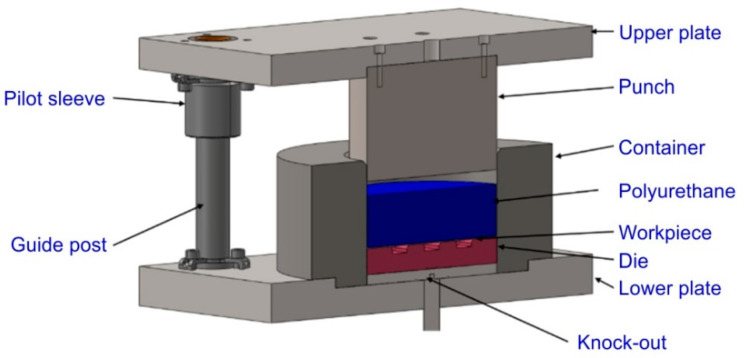
Stamping die for Guerin forming process of Hastelloy X sheets.

A special device (Figure 4) for rubber pad forming, also known as a Guerin box press, has been designed and fabricated.

**Figure 5:**
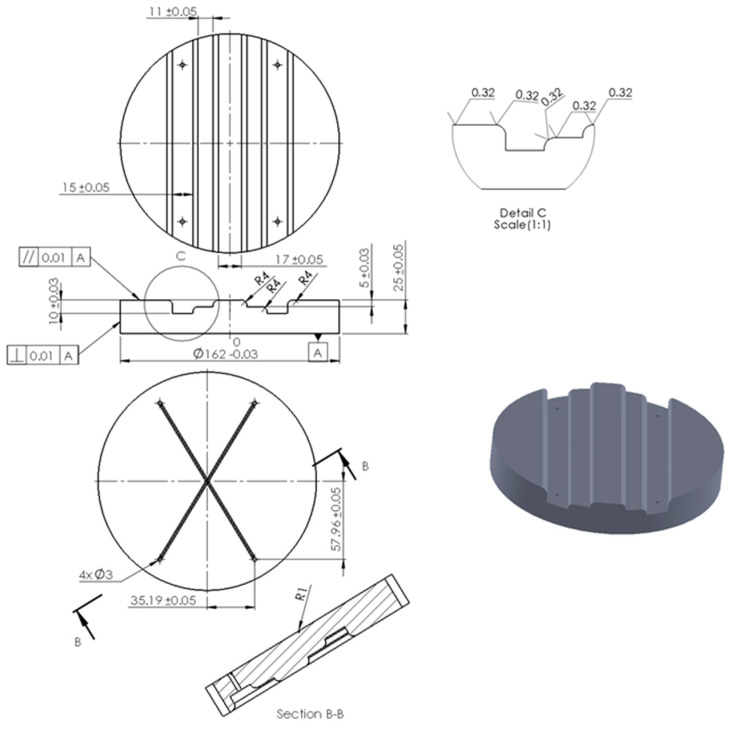
Working drawing of die.

The developed device was designed to allow for quick replacement of polyurethane inserts, a die, and a workpiece. Layered inserts of different hardnesses and configurations were used in the tests. The thickness of the elastomeric material layers was 10 mm. Figure 5 shows the working drawing of the die used for forming Hastelloy X sheet metal.

**Figure 6:**
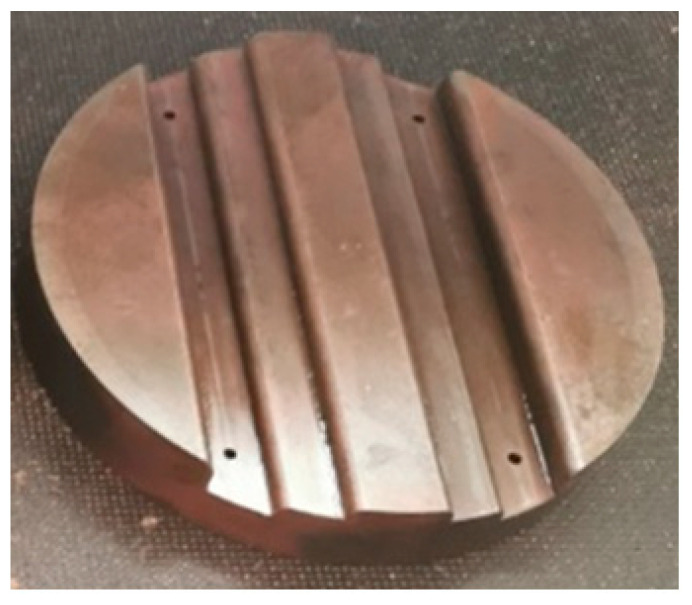
Die view.

The die shape (Figure 6) was designed to compare the effect of hardness of the elastomeric inserts in the case of forming a drawpiece consisted deep embossing. Additionally, the shape was developed so that the formed material underwent a two-stage process. The first stage consisted of forming until the sheet metal contacted the first level of embossing in a stamping die, and the second stage involved pressing the material into the deeper area of embossing in a stamping die. The die was made of tool steel heat treated to a hardness of 60 HRC.

**Figure 7:**
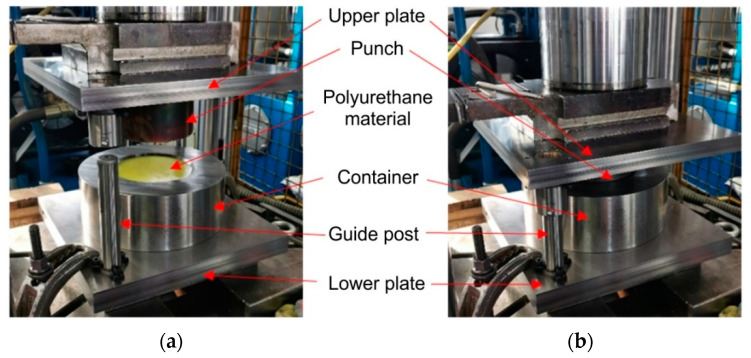
The polyurethane pad forming die (**a**) mounted on the press and (**b**) during forming.

The basic task of the experimental studies was to validate the results of numerical simulations. For this purpose, the forming device was mounted on a hydraulic press (Figure 7) with a pressure of 1471 kN. Venting channels (Figure 6) were used in the die, the purpose of which was to remove air from the forming zone, thus facilitating the filling of the die zone by the sheet pressed with PU inserts. To reduce friction in the processing zone, H-336 grease (BGM Molydal Sp.j., Mirosławice, Poland) intended for sheet forming was used.

### 2.5. Methodology of 3D Scanning of Drawpiece Geometry

The Atos Core 200 (GOM) (Carl Zeiss GOM Metrology GmbH, Braunschweig, Germany) non-contact system was used to assess the deformation of the drawpieces. Based on the views of the drawpiece from different perspectives, the program generates three-dimensional models of the products with an accuracy of 17 × 10^−3^ mm. The greatest depth of the embossing in the drawpieces was measured from the upper flat surface of the drawpiece (reference surface) to the point with the greatest depression. In addition, based on the measurements, the wall thickness distribution of the drawpiece, surface flatness, and forming unevenness were determined. The flatness deviation was determined using the best fit function.

### 2.6. Finite Element-Based Numerical Modeling

**Figure 8:**
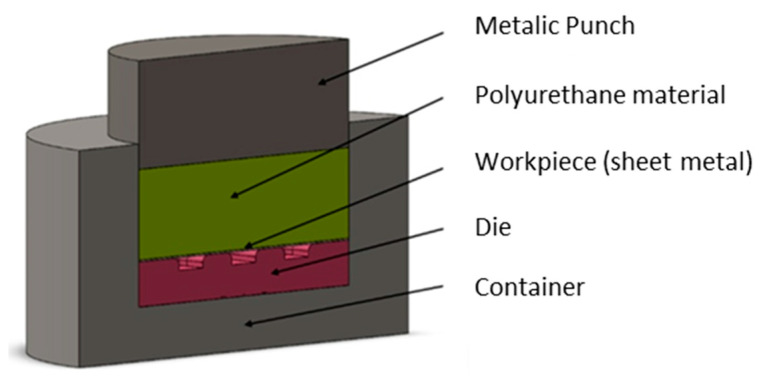
Cross-section of the 3D model of the tool.

FE-based Impetus Afea software (Impetusafea AB, version 8.1, Huddinge, Sweden) was used to model the Guerin box press forming of Hastelloy X drawpieces. The geometry of the container, punch, and die (Figure 8) corresponded to the experimental conditions.

**Figure 9:**
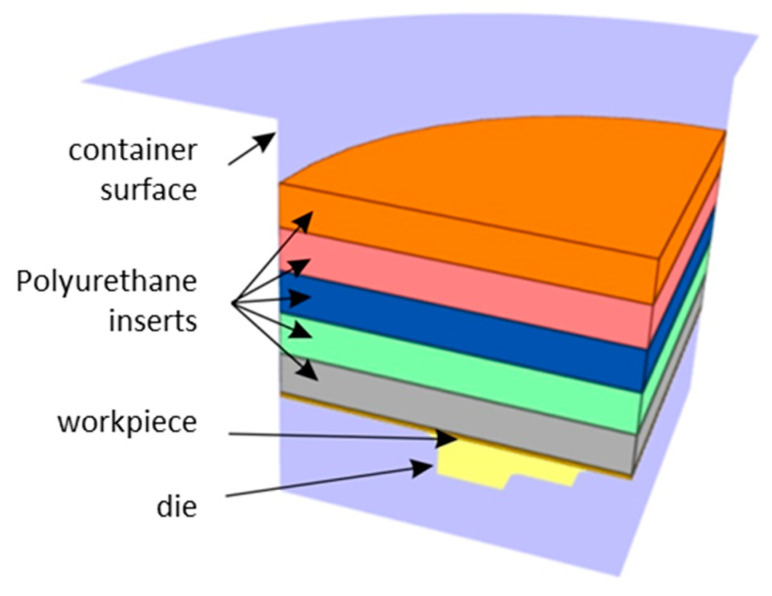
Geometric model of the Guerin box press forming.

Due to the symmetry of the forming process and to minimize the CPU time, the analyses were performed on the forming of a ¼ sheet model (Figure 9) with appropriate boundary conditions. The punch, die, and container were considered as non-deformable bodies. The sheet and polyurethane rubber inserts were deformable.

The material of Hastelly X alloy sheet was considered as elastic-plastic with parameters determined according to the procedures discussed in Section 2.2. The properties of elastomeric material were determined using the two-parameter Mooney–Rivlin model:

(4) $$\sigma_{1}=\frac{2C_{1}}{3}\left(2\lambda_{1}-\lambda_{2}-\;\lambda_{3}\right)-\;\frac{2C_{2}}{3}\left(\frac{2}{\lambda_{1}}-\frac{1}{\lambda_{2}}-\frac{1}{\lambda_{3}}\right)-\;\rho$$

(5) $$\sigma_{2}=\frac{2C_{1}}{3}\left(2\lambda_{2}-\lambda_{3}-\;\lambda_{1}\right)-\;\frac{2C_{2}}{3}\left(\frac{2}{\lambda_{2}}-\frac{1}{\lambda_{3}}-\frac{1}{\lambda_{1}}\right)-\;\rho$$

(6) $$\sigma_{3}=\frac{2C_{1}}{3}\left(2\lambda_{3}-\lambda_{1}-\;\lambda_{2}\right)-\;\frac{2C_{2}}{3}\left(\frac{2}{\lambda_{3}}-\frac{1}{\lambda_{1}}-\frac{1}{\lambda_{2}}\right)-\;\rho$$

(7) $$\rho =-K\varepsilon_{v}$$

where λ_1_–λ_3_ are the eigenvalues of the Cauchy tensor, σ_1_–σ_3_ are the principal stresses, ε_v_ is the volumetric strain, ρ is pressure and K, C_1_, and C_2_ are the material constants determined experimentally.

The values of material parameters (Table 2) of the polyurethane rubber with different hardnesses were determined using numerical simulations of compression of cylindrical samples.

The simulations were carried out in such a way as to select the optimal values of material parameters of elastomeric material ensuring the agreement of numerical results with experimental results of compression of cylindrical samples.

**Figure 10:**
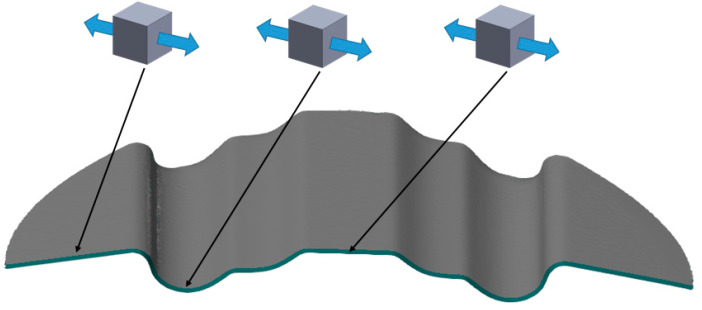
Cross-section of the drawpiece with stress states marked in specific areas.

Numerical simulations were performed for configurations of elastomeric inserts with the same hardness and configurations containing different properties of elastomeric material inserts. Figure 10 shows the cross-section of a drawpiece made using a die with two-stage cavities, showing the stress distribution in specific areas of the drawpiece.

The die shape was designed so that the dominant state was the bending of the material. The use of this shape was intended to verify the forming system during the bending tests of the drawpiece of a complex geometry. The stress state (uniaxial tension) is the same regardless of the location in the formed part and is perpendicular to the formed embossing. This tool shape was intended to verify the curvature of the face of the formed component since the die is intended to induce deflection of the sheet metal surface during the forming.

## 3. Research Results and Discussion

### 3.1. Mechanical Properties of Hastelloy X Sheets

**Figure 11:**
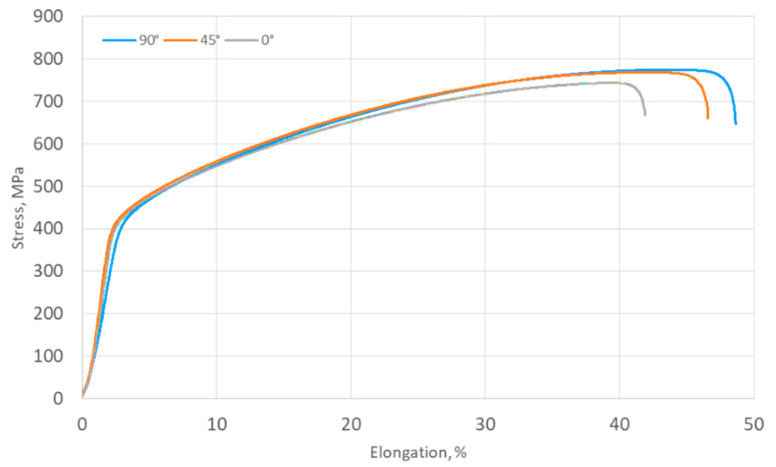
Selected stress–strain curves for Hastelloy X specimens oriented at 0°, 45°, and 90° relative to the sheet rolling direction.

Figure 11 shows selected stress–strain curves of Hastelloy X alloy sheets obtained for three specimens’ orientations relative to the rolling direction of the sheet metal.

All samples showed elongation exceeding 40%. The highest average elongation of 41.9% was observed for samples cut along the rolling direction of the sheet. On the other hand, samples cut perpendicular to the rolling direction of the sheet showed an average elongation of 48.6%. Table 3 shows a detailed quantitative summary of the average results obtained in uniaxial tensile tests of Hastelloy X alloy sheets.

The average values of the uniaxial tensile strength (UTS), yield stress (YS), and elongation (A) from all three orientations of the samples are 763.7 MPa, 401.6 MPa, and 45.9%, respectively, calculated as a weighted average, with a weight of 2 for the 45 orientations. The results clearly indicate the anisotropic features of the tested sheet, with the highest values obtained for an orientation of 90°. The difference between the highest and the lowest recorded values of UTS, YS, and A are 31.1 MPa, 9.4 MPa, and 6.7%, respectively. The average values of the normal anisotropy coefficient and the in-plane anisotropy coefficient are r = 1.17 and Δr = −0.60. The value of the normal anisotropy coefficient r = 1.17 allows us to estimate that the sheet will not thin significantly during forming, which allows it to be used for deep drawing. A negative value of the plane anisotropy coefficient means that during the conventional deep drawing process, there will be a tendency to form “ears” in the direction of 45° to the rolling direction of the sheet.

To sum up the results of the Erichsen sheet metal drawability tests (Table 4), it can be stated that the Hastelloy X alloy sheet is characterized by an average maximum drawing force of 38.4 kN, an average Erichsen index (IE) of 10.7 mm, a tensile strength of 640 MPa and an elongation of 39.9%.

### 3.2. Compressive Properties of Polyurethane Rubber

**Figure 12:**
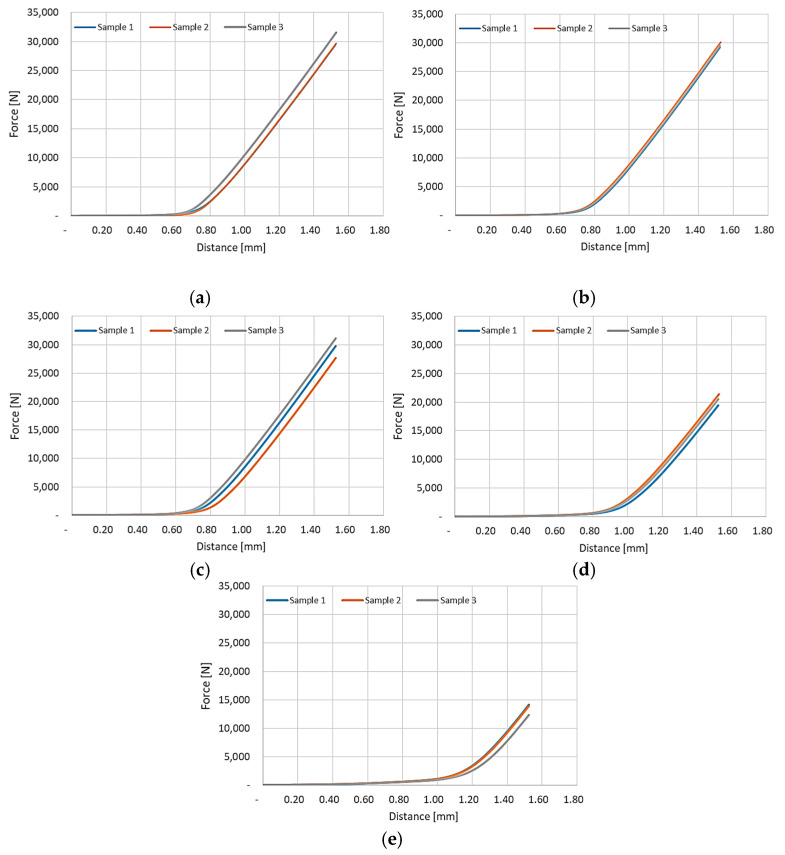
Results for the volumetric compression test of the polyurethane materials with hardness: (**a**) 50 ShA; (**b**) 60 ShA; (**c**) 70 ShA; (**d**) 80 ShA; and (**e**) 90 ShA.

Figure 12 presents the results of the volumetric compression tests for polyurethane rubbers of different hardness.

Analysis of the results allows determining the maximum force necessary to compress the sample in the Guerin box press forming by 1.5 mm at the level of 31,500 N, 29,200 N, 31,100 N, 21,400 N, and 14,100 N for samples with hardness of 50 ShA (Figure 12a), 60 ShA (Figure 12b), 70 ShA (Figure 12c), 80 ShA (Figure 12d), and 90 ShA (Figure 12e), respectively. The values of the maximum force for the three tested samples are the most similar for samples with a hardness of 60 ShA (Figure 12b). When testing the remaining samples, the largest difference was approx. 3600 N and 2000 N was recorded when testing samples with hardnesses of 70 ShA (Figure 12c) and 80 ShA (Figure 12d), respectively.

Comparing the results obtained for polyurethane rubber with different hardnesses, differences were noticed in the initial phase of the graphs. With the increase in material hardness, the initial compression force slowly rises, and then there is a transition to the volumetric compression phase. This means that with the increase in the hardness of the elastomeric material, the force exerted on the surface of the compressed material in the initial phase of the process increases. The differences in the maximum recorded force result from the application of an initial force of 10 N. This force is used to compensate for the friction resulting from the design of the stamping tool. Despite the use of the minimum initial force, it significantly influenced the results obtained. However, its use was necessary to make the measurement results comparable.

### 3.3. Results of Numerical Simulations

**Figure 13:**
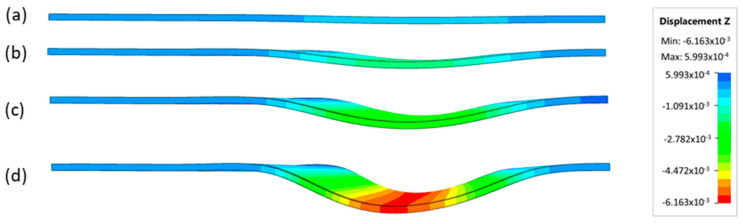
The successive steps of forming process for the following numerical calculation progress: (**a**) 20%; (**b**) 40%; (**c**) 60%; and (**d**) 90%.

Figure 13 shows the results of sheet metal deformation in the subsequent stages of numerical simulation.

Table 5 shows the simulation results of all variants of polyurethane insert configurations for Hastelloy X material.

The results are presented in the form of a perpendicular and cross-sectional view, which allows for an easier assessment of the obtained forming effects. The last column shows the maximum measured forming depth, which was the main parameter analyzed when comparing the tested configurations of polyurethane inserts. The forming depth was selected as the most important result included in the analyses, due to the clear differences in the individual combinations of polyurethane inserts and the possibility of easy validating the value obtained in numerical modeling with the results determined experimentally.

**Figure 14:**
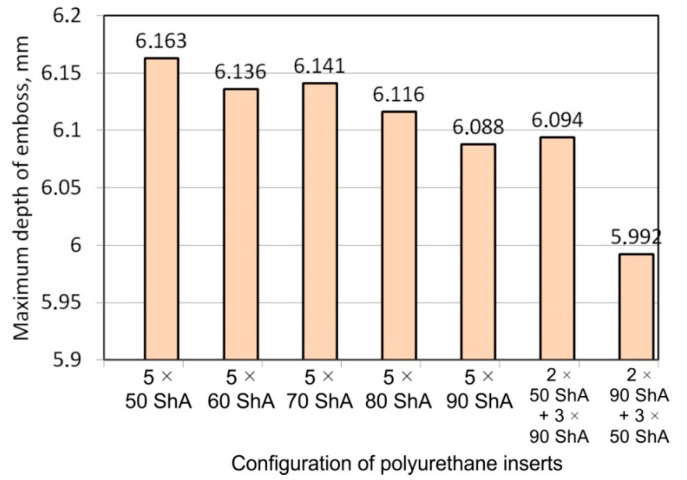
Influence of the configuration of polyurethane inserts on the maximum depth of embossing.

Figure 14 shows a graph comparing the depth of embossing for individual variants of polyurethane hardness during forming of apart from Hastelloy X material.

The best effect (the greatest depth of embossing) was achieved for polyurethane inserts with a hardness of 50 ShA. The value of the forming depth of embossing was 6.163 mm. For the polyurethane material variant containing two inserts of 90 ShA hardness and three inserts of 50 ShA hardness, the least favorable result of 5.992 mm was obtained. The difference between the analyzed variants is 0.171 mm. The percentage difference between the obtained results is 2.8% in relation to the depth of embossing obtained for the worst combination (3 × 50 ShA + 2 × 90 ShA).

Analysis of the results presented in Figure 14 allows us to state that in the case of elastomeric material consisting of inserts of the same hardness, there is a tendency to reduce the forming depth with the increase in the hardness of the polyurethane material. A comparison of the effects of the forming depth of the configuration of inserts of the same hardness showed differences at the level of 1.2%. Combinations of elastomeric materials assuming the use of two different hardnesses of polyurethane inserts allowed us to obtain smaller depths of embossing than for polyurethane inserts with a hardness of 50 ShA. In the case of using polyurethane inserts with a hardness of 90 ShA, a slight deflection of the formed material in the vertical direction occurred on the die surface. The deflection value was 0.03 mm. Such a small value is not subject to analysis due to the fact that it is impossible to measure the sheet deflection in the real forming conditions due to external factors, mainly the surface quality and deviations in the thickness of the formed sheet. No significant changes in the thickness of the formed sheets were observed. The numerically simulated thickness of the Hastelloy X sheet was 0.996 mm.

Among the analyzed configurations of the polyurethane inserts, the best variant was the one in which five inserts with a hardness of 50 ShA were used. Based on the results obtained during the numerical simulations, extreme variants of the polyurethane material configurations were selected for the experimental tests. Table 6 presents the selected combinations.

Variant A denotes the polyurethane hardness variant giving the least favorable results of the embossing depth. Variant B is the system that produced the deepest embossing in drawpieces. Simulations were also carried out for the target forming force, which was 100 Mg of the press force on the polyurethane inserts. Despite such a high force, there were no significant differences in the deformation of the sheets depending on the hardness of the polyurethane inserts.

### 3.4. Experimental Results

Table 7 presents a summary of the results of the Guerin forming process of drawpieces obtained by forming on a hydraulic press with a press force of 100 Mg, depending on the arrangement and hardness of the polyurethane inserts.

The table contains photographs of the samples, a view of the 3D model, and the measured maximum forming depth of embossing for each variant. For the variant with two inserts of hardness 90 ShA and three inserts of hardness 50 ShA, the maximum forming depth of embossing was 10.01 mm, and for the variant in which five inserts of hardness 50 ShA were used, this value was equal to 10.13 mm.

**Figure 15:**
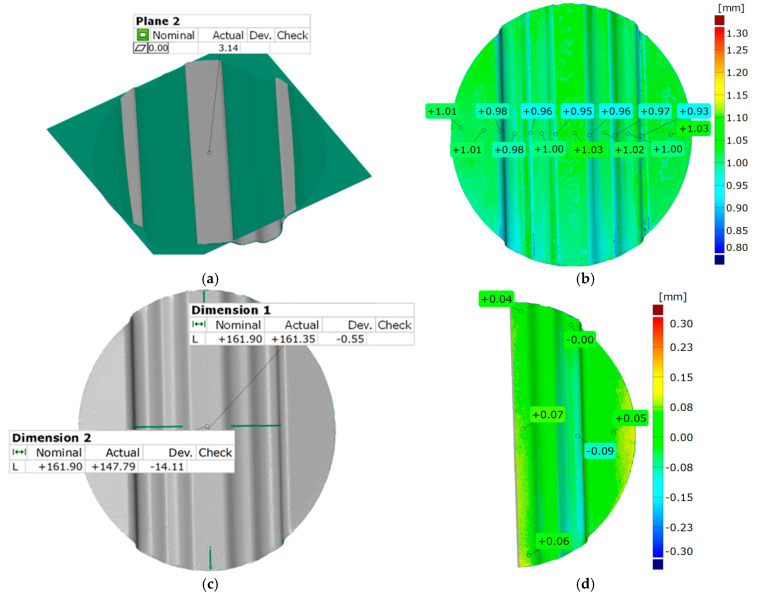
Geometric parameters of the Hastelloy X alloy drawpiece formed using the configuration of polyurethane inserts 2 × 90 ShA + 3 × 50 ShA: (**a**) surface flatness; (**b**) thickness change; (**c**) change of external dimensions of the disc; (**d**) forming uniformity.

**Figure 16:**
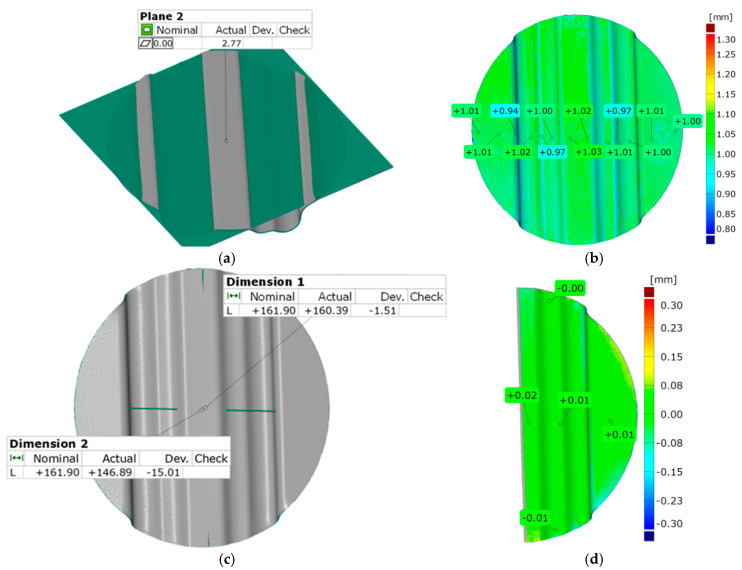
Geometric parameters of the Hastelloy X alloy drawpiece formed using the configuration of polyurethane inserts 5 × 50 ShA: (**a**) surface flatness; (**b**) thickness change; (**c**) change of external dimensions of the disc; (**d**) forming uniformity.

A difference in the depth of embossing between the used variants of the polyurethane inserts equal to 0.12 mm was noted. Despite such a small difference, the trend was maintained, and in each case, the variant of inserts selected as the best did not achieve a lower forming depth of embossing than the variant selected as the worst. The results of the geometry measurement of the drawpiece, obtained by pressing with two polyurethane inserts of hardness 90 ShA, three polyurethane inserts of hardness 50 ShA, and five polyurethane inserts of hardness 50 ShA, are presented in Figure 15 and Figure 16, respectively.

In the case of surface flatness (Figure 15a and Figure 16a), there was a difference of 0.37 mm for both configurations of polyurethane inserts. For the variant 2 × 90 ShA 3 × 50 ShA the surface flatness was 3.14 mm, and for the 5 × 50 ShA configuration the surface flatness was 2.77 mm.

The wall thickness distributions of the drawpieces are shown in Figure 15b and Figure 16b. In the case of the 2 × 90 ShA + 3 × 50 ShA configuration, a fairly uniform thickness distribution occurs. The minimum measured sheet thickness value was 0.95 mm, and the largest was 1.03 mm. The difference between the maximum and minimum values is 0.08 mm, which gives a maximum wall thinning of about 6%. In the case of the 5 × 50 ShA configuration, the thinning is very similar, no significant changes in wall thickness were observed in relation to the 2 × 90 ShA + 3 × 50 ShA variant.

The change in the diameter of the formed disc, visible in Figure 15c and Figure 16c, for the 2 × 90 ShA + 3 × 50 ShA variant was −0.55 mm longitudinally to the direction of the formed embossing and −14.11 mm in the transverse direction. These values for the 5 × 50 ShA configuration were −1.51 mm and −15.01 mm, respectively. The deviations of the forming uniformity (Figure 15d and Figure 16d) are much greater for the 2 × 90 ShA + 3 × 50 ShA polyurethane insert variant and amount to −0.09 and +0.07 mm (Figure 15d). The same deviations for the 5 × 50 ShA configuration are −0.01 and +0.02 mm (Figure 16d).

**Figure 17:**
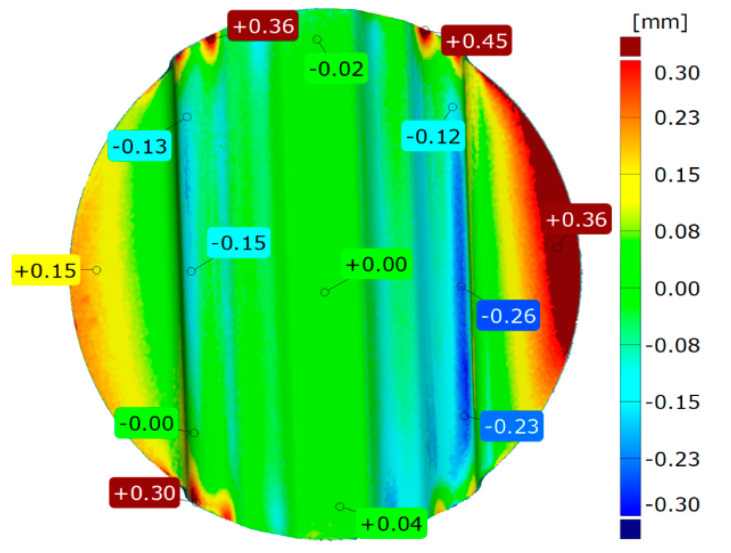
Difference between drawpieces formed using polyurethane insert configurations of 2 × 90 ShA + 3 × 50 ShA and 5 × 50 ShA.

Figure 17 shows a comparison of 3D scans of measured drawpieces obtained by using different polyurethane inserts.

The obtained results show greater possibilities of obtaining deeper embosses using the 5 × 50 ShA polyurethane insert configuration. The differences in depths of embosses range from −0.12 mm to −0.23 mm. In the case of the 5 × 50 ShA configuration, a greater flatness of the front surface of the drawpiece was obtained. For the wall thickness and forming uniformity, the results are comparable for the analyzed insert configurations.

## 4. Summary and Conclusions

This work presents the results of experimental and numerical investigations on the effect of polyurethane hardness on the formability of Hastelloy X sheet metals in the Guerin process. The aim of the numerical studies was to determine the most favorable and worst configurations of polyurethane inserts. The geometry of the drawpieces was evaluated using a non-contact metrology 3D Atos Core 200 scanner. The flatness of the drawpieces, sheet thinning, and non-uniformity of forming were evaluated. Based on the experimental and numerical results, presented in this work, the following conclusions can be drawn:

The tested sheets were characterized by an average elongation of 45.7%. The highest values of basic mechanical parameters, i.e., the yield strength, ultimate tensile strength, and elongation, were recorded for samples cut transversely to the rolling direction of the sheet. Deep-drawing characteristics of Hastelloy X alloy sheets were confirmed by the value of the plane anisotropy coefficient r = 1.17.

The results of numerical analyses, validated experimentally, proved that the hardness of polyurethane inserts has a key influence on the character of deformation of Hastelloy X sheet metals in the Guerin process. It was found that the variant of the stamping tool containing inserts of the same hardness of 50 ShA ensured the formation of the deepest embossing. For the variant containing two inserts of hardness 90 ShA and three inserts of hardness 50 ShA, the maximum forming depth was 10.01 mm. On the other hand, for the variant in which 5 inserts of hardness 50 ShA were used, this value was equal to 10.13 mm.

The resistance to deformation of the polyurethane material depends on its hardness and is much greater when a lower forming pressure is used. The configuration and hardness of polyurethane inserts should be selected depending on the shape of the die.

Volumetric compression tests showed that in the case of polyurethane rubber with a hardness of 50–60 ShA, the differences in maximum compressive forces are small, while for the remaining hardnesses of 70–90 ShA, significant differences in the value of these forces are visible. The high hardness of the elastomer material allows the polyurethane inserts to transfer greater force to the sheet metal surface in the initial stages of forming. The lower hardness of the elastomer material, however, allows the sheet metal to fill the die outline more precisely with lower pressure exerted on the elastomer.

Increasing the sheet forming force (up to 100 Mg) significantly reduces the influence of polyurethane insert hardness on the accuracy of die-shape reproduction. A difference in the depth of embossing between the polyurethane insert variants used was noted as equal to 0.12 mm.

The use of polyurethane inserts of different hardnesses determines the different wear of the elastomeric material on the contact surface with the formed sheet metal. The advantage of using multi-layer elastomeric tools is the possibility of replacing individual inserts in the event of their wear and greater flexibility of the production process resulting from the possibility of configuring inserts with different characteristics.

Surface flatness measurements of the drawpieces indicated a difference in flatness of 0.37 mm between the two elastomeric material configurations. For the 2 × 90 ShA + 3 × 50 ShA variant, the surface flatness was 3.14 mm, and for the 5 × 50 ShA variant 2.77 mm. Large flatness deviations result from the elastic-plastic properties of the Hastelloy X alloy and the shape of the drawpieces, which contain embosses of a shape that causes uniaxial tensile stresses.

A limitation of the Guerin forming process using elastomeric tools is the inability to perform the forming process of difficult-to-form alloys in hot forming conditions.

## Figures and Tables

**Table 1:** Chemical composition of the Hastelloy X alloy (wt.%) [27].

Ni	Cr	Fe	Mo	W	C	Mg	Si	P	S	Al	Ti	Co	Cu
Bal.	20.5–23	17–20	8–10	0.2–1	0.05–0.15	max. 1	max. 1	max. 0.04	max. 0.03	max 0.5	Max 0.15	0.5–2.5	max. 0.5

**Table 2:** Material coefficients of polyurethane material.

Polyurethane Hardness, ShA	Coefficient *C*_1_, Pa	Coefficient *C*_2_, Pa	Coefficient *K*, Pa
50	0.3 × 10^6^	0.15 × 10^6^	4.0 × 10^9^
60	0.59 × 10^6^	0.19 × 10^6^	4.1 × 10^9^
70	0.6 × 10^6^	0.2 × 10^6^	4.2 × 10^9^
80	1.6 × 10^6^	0.11 × 10^6^	4.8 × 10^9^
90	2.1 × 10^6^	0.1 × 10^6^	4.8 × 10^9^

**Table 3:** Selected mechanical properties of Hastelloy X alloy sheets (average values of three measurements for each sample orientation).

Sample Orientation	Uniaxial Tensile Strength UTS MPa	Yield Stress YS, MPa	Elongation A, %
0°	743.3	396.3	41.9
45°	768.5	402.2	46.5
90°	774.4	405.7	48.6
Mean	763.7	401.6	45.9

**Table 4:** Results of Hastelloy X sheet metal drawability tests.

Sample Number	Maximum Force, kN	Erichsen Index, mm	Uniaxial Tensile Strengths, MPa	Elongation, %
1	37.79	10.5	632.41	39.39
2	38.96	10.8	648.63	40.5
3	38.41	10.7	638.90	39.79

**Table 5:** Results of maximum depth of embossing in Hastelloy X drawpieces for individual hardness variants of the polyurethane rubber.

Hardness of Elastomeric Material	Numerical Results	Maximum Depth of Emboss, mm
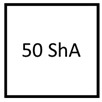	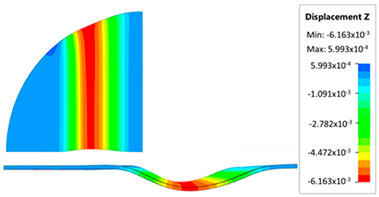	6.163
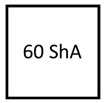	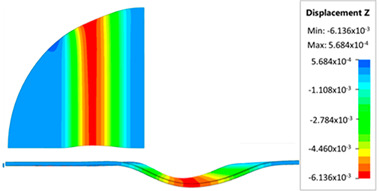	6.136
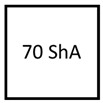	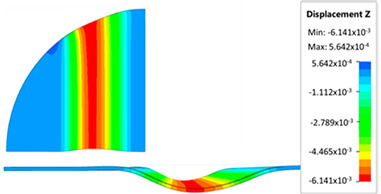	6.141
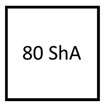	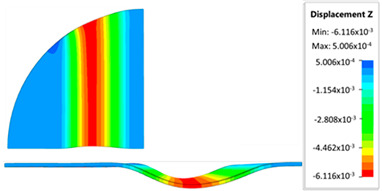	6.116
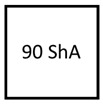	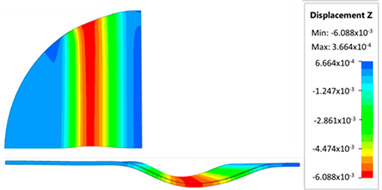	6.088
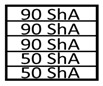	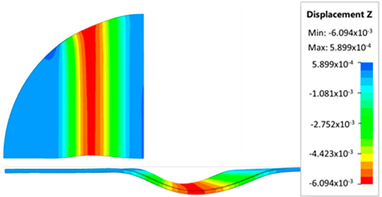	6.094
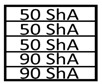	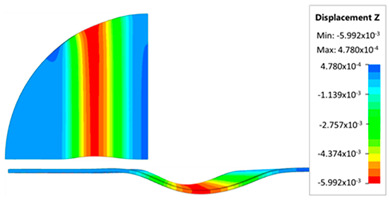	5.992

**Table 6:** Configuration variants of polyurethane inserts with different hardnesses intended for experimental tests.

Variant	A	B
Configuration of polyurethane inserts	2 × 90 ShA + 3 × 50 ShA	5 × 50 ShA

**Table 7:** Summary of the results of experimental tests.

Configuration of Polyurethane Inserts	Photo of the Drawpiece	3D Scan of Drawpiece	Maximum Depth of Embossing, mm
5 × 50 ShA	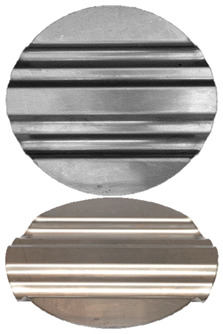	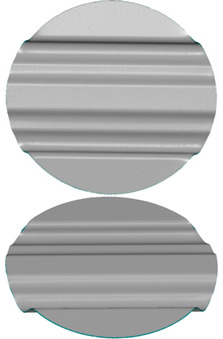	10.13
2 × 90 ShA + 3 × 50 ShA	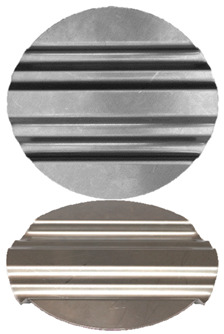	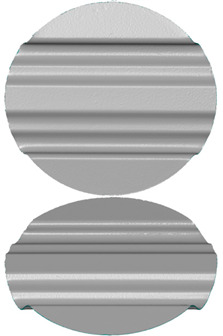	10.01

## Data Availability

Data are available from the first author and can be shared with anyone upon reasonable request.
